# Correlated Subjects: Relational Ethics and Veterinary Legal Accountability in Animal-Assisted Interventions

**DOI:** 10.3390/ani16010092

**Published:** 2025-12-29

**Authors:** Paola Fossati

**Affiliations:** Department of Environmental Science and Policy, Università degli Studi di Milano, 20133 Milan, Italy; paola.fossati@unimi.it

**Keywords:** relational ethics, vulnerability, animal law, animal-assisted interventions, veterinary bioethics, institutional accountability

## Abstract

The ethical and legal governance of Animal-Assisted Interventions (AAIs) remains conceptually and normatively fragmented. Although animals engaged in therapeutic, educational, and assistive activities make valuable contributions to human well-being, they continue to be defined by law as property or welfare objects, despite their meaningful yet limited forms of relational participation within structured human-controlled environments. This perspective obscures their context-dependent responsiveness and their institutional embeddedness. The present paper addresses this gap by adopting a normative and interdisciplinary approach grounded in relational legal theory and vulnerability scholarship. The concept is developed by drawing on Jennifer Nedelsky’s notion of relational autonomy and Martha Fineman’s theory of universal vulnerability. This results in the conceptualisation of AAI animals as correlated subjects: beings whose ethical and legal significance derives from the relationships and institutional contexts that shape their participation. The analysis identifies weaknesses in current medico-legal practices that frame veterinary certification and welfare assessment as static technical acts, ignoring their relational and systemic dimensions. The paper puts forward a relational ethical–legal framework for Animal-Assisted Interventions, centred on relational vulnerability, context-sensitive oversight and continuous institutional accountability. A number of practical recommendations are put forward, including the introduction of renewable ethical licences, inter-institutional monitoring and the establishment of multidisciplinary oversight mechanisms. By redefining animals’ normative status through relational ethics, in alignment with the interconnected human, animal, and environmental dimensions emphasized by the One Welfare principles, the study advances a shift from welfare-based protection toward a model of justice grounded in interspecies interdependence and institutional responsiveness.

## Simple Summary

Animals used in assistive, educational, or therapeutic programmes—also referred to as Animal-Assisted Interventions (AAIs)—play a pivotal role in the advancement of human well-being. However, laws and ethical rules often treat these animals merely as property or tools. The present article puts forward a novel approach to comprehending their status. It employs recent concepts in the fields of ethics and law to argue that AAI animals should be recognised as correlated subjects, defined as living beings whose well-being and performance are contingent on their relationships, institutional contexts, and interactions with humans. The present paper examines the question of how veterinarians, handlers, and organisations share responsibility for the welfare of animals. It further suggests several practical measures, including the introduction of continuous welfare monitoring, time-limited certification, and the establishment of institutional accountability. By linking veterinary ethics, legal responsibility, and the One Welfare approach, the study advances a novel, more compassionate, and just framework for human–animal partnerships in AAI settings.

## 1. Introduction

The legal and ethical status of nonhuman animals has undergone significant scrutiny over recent decades, particularly in light of their increasingly complex and institutionalised roles within human society. In this context, the animals engaged in therapeutic, educational, and assistive programmes, commonly referred to as Animal-Assisted Interventions (AAIs) (In this article, Animal-Assisted Interventions refer to structured therapeutic, educational, or assistive programmes in which trained animals work with qualified professionals to support human well-being within institutional settings), occupy a distinctive position: they perform specialised therapeutic, educational and supportive tasks of recognised social importance, yet they remain legally framed as property or instruments. This disjunction between social function and normative status exposes a profound conceptual gap within contemporary governance [1,2].

Traditional legal dichotomies between “persons” and “things”, subjects and objects, or agents and recipients, fail to capture the situated reality of working animals, whose participation in human institutions reflects context-dependent responsiveness, relational involvement, and forms of dependency. The persistence of these binaries is increasingly untenable as ethology, behavioural science, and moral philosophy reveal the cognitive complexity and emotional sentience of many species [3,4]. At present, however, the prevailing normative guidance in this field consists largely of professional guidelines, institutional protocols, and other soft-law instruments (Examples drawn from the Italian context appear more frequently because Italy is one of the few jurisdictions with national guidelines specifically dedicated to AAIs, and made binding by the law, and it is the regulatory context with which the author is most acquainted. The broader argument developed in this article, however, concerns structural patterns that extend well beyond the Italian system). The existence of coherent statutory frameworks that specifically regulate Animal-Assisted Interventions remains limited. In this article, the terms “legal frameworks” and “legal infrastructure” refer primarily to statutory or regulatory provisions that directly govern Animal-Assisted Interventions, as distinct from non-binding professional guidelines that, at present, provide much of the normative guidance in the examined field, both at international and national levels. Relational legal theory offers a promising corrective to this conceptual inadequacy. Pioneered by scholars such as Jennifer Nedelsky [5] and Martha Fineman [6], this challenges the liberal idea of legal subjectivity as an abstract attribute of the autonomous, self-sufficient individual. Instead, it argues that personhood, autonomy and capacity are not inherent traits, but emerge from networks of relationships and dependencies. This signifies that autonomy and personhood are not innate qualities; rather, they are facilitated by relationships and institutional contexts that provide support. Although developed with humans in mind, these frameworks illuminate the conditions under which animals, too, are constituted as actors within relational systems of care, regulation, and work [7,8]. Importantly, they do not attribute autonomous agency to animals; rather, they highlight their constrained yet observable forms of responsiveness within human-mediated settings.

Building on this insight, the present paper proposes an ethical-legal paradigm that transcends property-based models and recognises AAI animals as “correlated subjects”: beings whose normative significance stems from their relational roles within human institutions. In this paper, the expression ‘correlated subjects’ refers to animals whose ethical and legal significance arises from their interdependence with human and institutional counterparts. The term emphasises the relational nature of both their agency and normative status, indicating that these are co-defined through relationships rather than possessed individually. It also conveys the reciprocal constitution of responsibilities that characterises Animal-Assisted Interventions (AAIs). This concept neither anthropomorphises animals nor attributes to them autonomy in the human sense. Rather, it acknowledges that animals involved in structured AAI relationships exhibit forms of responsiveness and participation that warrant ethical and legal consideration. Their agency is relational rather than individual: they act, respond, and adapt within institutional power structures that both enable and constrain their well-being. This has far-reaching implications for policy, regulation, and veterinary practice.

The argument is based on the premise that legal and ethical responsibility must be understood as distributed and dynamic. In practice, the conditions under which animals perform therapeutic work are co-produced by veterinarians, handlers and institutions, yet the mechanisms by which accountability is defined remain poorly defined. A pertinent example of this phenomenon is that of veterinary certification, which has been frequently observed to be regarded as a static technical authorisation rather than a relational judgment necessitating continual reassessment. This finding unveils a medico-legal blind spot that mirrors Fineman’s [6] critique of law’s inability to address vulnerability as a universal and institutional condition.

In order to address these shortcomings, the paper integrates relational and vulnerability theory into a model of veterinary and institutional accountability suited to AAI contexts.

The paper calls for the development of adaptive frameworks, such as ethical licensing, continuous welfare monitoring, and inter-institutional databases. The aim is to replace episodic, owner-centred regulation with systemic oversight grounded in care and transparency.

Methodologically, the analysis is normative and interdisciplinary. As a conceptual inquiry, the paper does not rely on empirical datasets. References to current practices (e.g., diffusion of responsibility or absence of review mechanisms) draw on published literature in veterinary ethics, AAI guidelines, and comparative regulatory studies rather than statistical evidence. In particular, the present text synthesises relational jurisprudence, veterinary bioethics, and policy studies in order to articulate an integrated ethical–legal framework. The objective is not empirical description per se; rather, it is concerned with conceptual innovation: to situate AAI animals within a relational architecture of justice capable of reflecting their lived interdependence with human actors and institutions.

The remainder of this article outlines the theoretical foundations of relational autonomy and vulnerability (Section 2), examines the legal and medico-ethical gaps affecting Animal-Assisted Interventions (Section 3), and proposes a relational accountability model with corresponding regulatory implications (Section 4).

### Methodological Note

The present study adopts a conceptual and normative methodology grounded in interdisciplinary bioethical analysis. Rather than utilising empirical data or quantitative measurement, the argument is developed through a process of conceptual synthesis that integrates relational jurisprudence, veterinary ethics, and vulnerability theory. The analysis draws upon principles from analytic jurisprudence, as well as critical strands of bioethical scholarship. The aim is to expose normative tensions in existing welfare regimes and to outline a framework that may be adapted to institutional practice.

The selection of sources has been made with a view to their relevance to relational legal theory, feminist care ethics, and emerging discourses in One Welfare and multispecies justice. This method allows for the systematic mapping of concepts, such as autonomy, vulnerability, and accountability, across legal, ethical, and veterinary domains. The analysis is thus interpretive and policy-oriented: it seeks to generate a coherent normative architecture rather than to produce empirical generalisations. By clarifying the conceptual and relational underpinnings of animal participation in therapeutic work, the study provides a theoretical basis for practical governance reforms in Animal-Assisted Interventions (AAIs).

## 2. Theoretical Framework: Relational Legal Theory and Its Relevance to Nonhuman Animals

### 2.1. Relational Autonomy and the Situated Self

Relational legal theory challenges the liberal assumption that legal personhood derives from self-sufficiency, rationality, or autonomy conceived as independence. Within this traditional framework, beings who do not meet the threshold of autonomy and rational agency are categorised as legal “things” rather than “persons”, thereby excluding most nonhuman animals from substantive legal recognition [1,2] and reducing them to the status of property or welfare objects, disregarding the relational contexts in which their capacities develop.

Nevertheless, over the course of the last two decades, a new framework has emerged, challenging the adequacy and fairness of this binary structure. This approach is based on the premise that legal subjectivity arises from the network of relationships, dependencies, and institutional roles that a being occupies. These developments require a rethinking of agency, responsibility, and membership in legal contexts, with significant implications for the legal status of non-human animals.

Within the paradigm of relational legal theory, Jennifer Nedelsky [5], a jurist and political theorist, has proposed a compelling model of relational autonomy to explain human legal subjectivity. The model posits that autonomy and agency are co-constituted within webs of relationships, dependencies, and institutions. The concept of autonomy, as outlined in this account, is not regarded as an innate, pre-legal attribute, but rather as a social and institutional achievement supported by relationships of care, recognition and support. Consequently, the legal system’s role is not solely to safeguard individual autonomy but also to establish and preserve the frameworks that enable self-determination.

When applied to the context of human–animal interactions, Nedelsky’s model provides a critical framework for re-evaluating the ethical and legal status of animals involved in structured human–animal relationships, such as those within Animal-Assisted Interventions (AAIs). These animals are selected, trained, and evaluated to operate within organised systems of care and responsibility. Understanding how autonomy functions within such relational settings requires turning to Nedelsky’s account of autonomy itself. In her view, autonomy is not merely a matter of individual independence; rather, it is a capacity that emerges through supportive relationships and enabling institutional conditions. In essence, individuals, human or non-human, can act autonomously only insofar as the relationships surrounding them and the institutional environment engender the conditions that make meaningful action possible. To provide an illustration, the capacity of a child or elderly person to exercise autonomy is contingent upon the provision of the necessary support from caregivers and institutions. Within Animal-Assisted Interventions, animals’ autonomy is therefore relational rather than independent or reflective: it arises through cooperation, mutual responsiveness, and embeddedness within human institutions. It should be emphasised that such autonomy remains highly constrained by human-controlled environments and does not resemble independent choice or voluntary participation.

This perspective does not entail anthropomorphism; rather, it reflects animals’ lived realities more accurately than the conventional dichotomy of freedom versus control [9]. Nedelsky’s observation that “relations of dependence and hierarchy can be structured to foster rather than undermine autonomy” [5] (p. 46) is particularly relevant. Within the context of Animal-Assisted Interventions (AAIs), animals’ capacity to act responsively depends on institutional arrangements, encompassing selection, training, rest protocols, and welfare assessment, which can function either as enabling factors promoting well-being or as constraining factors that impede it. The recognition of these relational conditions allows autonomy to be understood as an experiential state of enabled participation rather than a marker of rational independence.

Extending Nedelsky’s framework to interspecies contexts highlights autonomy as multidimensional and context-specific. The ethical question, therefore, is not whether animals possess autonomy, but how institutions create (or fail to create) the conditions under which animals can display context-dependent forms of relational responsiveness. This approach provides a theoretical foundation for the attribution of legal and ethical significance to animals whose capacities are shaped by institutional dependence rather than diminished by it [7,8].

### 2.2. Universal Vulnerability and Responsive Institutions

Martha Fineman [6], an American jurist, legal theorist and political philosopher, proposes the theory of the vulnerable subject that provides a complementary and equally transformative perspective on legal and ethical personhood. Rejecting the liberal image of the autonomous, self-sufficient agent, Fineman argues that vulnerability is an inherent feature of embodied life. Far from denoting weakness, vulnerability signifies the shared susceptibility to harm, dependency and change that characterises both human and non-human existence. As such, justice cannot be grounded in the protection of independent individuals alone; it must instead ensure that institutions are designed to respond to the vulnerabilities that shape all lives [10].

Fineman’s framework reframes legal responsibility as responsive rather than merely reactive. The “responsive state” she envisions actively structures social, political and economic conditions that enable resilience, the capacity to adapt and flourish despite inevitable vulnerability. In this vision, equality and justice are achieved not by treating all individuals as the same, but by recognising differential needs and dependencies and by allocating institutional support accordingly.

Although Fineman’s work was developed to address human jurisprudence, its principles extend powerfully to the non-human domain. Animals, too, are embodied and dependent beings, whose vulnerabilities are often intensified by their human use and contexts of institutional subordination. Yet, in most legal systems, their vulnerability is treated as incidental rather than constitutive, addressing it through limited welfare provisions rather than a foundational condition that should structure ethical and legal responsibility [7,11]. This results in a protection model that is both episodic and individualistic, overlooking systemic factors, such as training intensity, workload, exposure, and institutional culture, that shape animal well-being within Animal-Assisted Interventions (AAIs).

A vulnerability-based approach shifts the legal focus from rights to needs and resilience. Rather than posing the question of whether animals meet the necessary thresholds of cognition or relational agency sufficient for rights-bearing status, this perspective examines how institutions can distribute care more equitably and minimise risk across species boundaries. This aligns with contemporary developments in relational animal ethics and multispecies justice [12,13], emphasising that vulnerability is not a moral deficiency but rather the shared basis for ethical obligations among human professionals involved in the intervention. Fineman’s concept of shared vulnerability also complements the One Welfare framework, which links human, animal, and environmental well-being as interdependent domains. In Animal-Assisted Interventions (AAIs), relational vulnerability manifests simultaneously in the animal’s welfare state, the human beneficiary’s condition, and the therapeutic environment in which both interact.

For veterinary and regulatory practice, this reorientation carries tangible implications. A responsive institutional framework would require that the welfare of animals in interventions be continuously monitored through adaptive mechanisms (e.g., time-limited certifications, reassessment protocols, and transparent data-sharing) akin to oversight structures used to protect human workers from occupational harm. In this way, Fineman’s principle of institutional responsiveness can be operationalised to ensure that animals’ vulnerabilities are recognised not as private concerns of handlers or owners, but as collective ethical and legal responsibilities distributed across systems of care and governance.

Ultimately, integrating Fineman’s theory of universal vulnerability into animal law and bioethics challenges the anthropocentric foundations of legal personhood. It demands that justice extend beyond formal equality to encompass relational equity: a system in which both human and non-human subjects are supported in their interdependent flourishing through responsive, accountable institutions.

### 2.3. Relational Paradigms and Gradated Legal Subjectivity

When considered together, the theories of relational autonomy and universal vulnerability converge on a shared critique of atomistic subjectivity and a commitment to the idea that agency and justice are relationally constituted. Both reject the binary opposition between autonomy and dependence, proposing instead a continuum of relational participation in which humans attribute normative significance to animals’ roles from their embeddedness within networks of care, responsibility, and institutional power.

Contemporary scholarship has increasingly extended this relational turn to non-human animals, challenging the anthropocentric boundaries that have long shaped legal and moral consideration. In their seminal work Zoopolis, Donaldson and Kymlicka [9] advanced a novel conceptual framework by proposing distinct models of citizenship for domesticated, wild, and liminal animals. This pioneering work positions domesticated animals, including those engaged in interventions, as members of interspecies communities who participate in shared practices and responsibilities, thereby reframing them as co-citizens rather than passive dependants.

In a similar vein, Saskia Stucki [8] advances the concept of graduated legal subjectivity, arguing that animals should be recognised not as full legal persons nor as mere property, but as entities possessing context-dependent legal capacities. Legal status, in this view, exists along a spectrum shaped by the relational and functional roles animals occupy within human institutions. To illustrate this point, the case of an assistance dog or therapy horse can be considered. These animals engage in relationships characterised by trust and expectation, while the human counterpart assumes the relevant caregiving responsibilities. These human responsibilities differ significantly from those observed in relationships with companion animals living in non-institutional contexts. Feminist legal theorist Maneesha Deckha [7] further broadens this relational paradigm by urging a posthumanist, care-based ethic that moves beyond the liberal humanist model of autonomy and rationality. Drawing on feminist care theory and posthumanism as a foundation, she emphasises relationality, affect, and dependency as foundational principles of justice. In this view, animals are not passive recipients of protection but ethical participants in interspecies relationships that shape shared forms of well-being and responsibility [14].

These perspectives collectively advance what may be termed a relational paradigm of legal subjecthood, which grounds recognition not in cognitive capacity or species membership, but in the lived realities of participation within interdependent relationships. The concept of “correlated subjects”, as used in this paper, refers to animals whose ethical and legal significance derives from their integration into institutional systems of care, education, or therapy. Such animals occupy neither a position of full autonomy nor one of mere domination; nor are they conceptualised as symmetrical partners. Instead, their roles reflect constrained forms of context-dependent responsiveness within settings shaped by human actors, and generate complementary obligations among veterinarians, handlers, and institutional providers.

Recognising AAI animals as correlated subjects thus provides a conceptual bridge between ethical theory and legal practice. It reframes veterinary and institutional responsibilities not as a matter of discretionary welfare but as structural components of justice. This shift encourages the law to move beyond the limiting dichotomy of “person versus property” and to acknowledge animals’ participation in relational frameworks that carry genuine ethical and juridical weight.

## 3. Legal Gaps and Veterinary Responsibility in the Context of Animal-Assisted Interventions

The regulation of Animal-Assisted Interventions (AAIs) remains fragmented and conceptually inconsistent across jurisdictions. Despite the increasing institutionalisation of AAIs, animals involved in these practices continue to occupy a legally ambiguous status, straddling the line between therapeutic instruments and co-working agents. This ambiguity gives rise to significant medico-legal and ethical consequences, particularly for veterinarians and institutions tasked with safeguarding animal welfare within human-centred systems.

### 3.1. Veterinary Responsibility and Medico-Legal Implications

Veterinary professionals occupy a pivotal position at the intersection of ethics, medicine, and law in AAI governance. Conventionally, the domain of veterinary responsibility has been delineated in technical terms, with a primary focus on the preservation of animal health and the issuance of certifications attesting to their fitness for specific work. Nevertheless, from relational and institutional viewpoints, this restricted conception is inadequate in comprehensively addressing the broader normative ramifications inherent to the veterinarian’s function. Within the context of Animal-Assisted Interventions (AAIs), veterinarians are tasked with the responsibility of evaluating physical health alongside behavioural indicators such as stress signals, fatigue, avoidance, withdrawal cues, and changes in social responsiveness, and with assessing, albeit indirectly, an animal’s capacity to sustain therapeutic relationships within institutional settings [15].

Currently, such assessments are largely regarded as static, individualised endorsements. Once issued, they remain valid indefinitely unless a welfare crisis occurs. Such practices obscure the dynamic, context-sensitive nature of animal well-being and the medico-legal accountability associated with certification. For instance, when an animal previously deemed fit begins to exhibit signs of chronic stress or fatigue, there is rarely a clear legal mechanism to allocate responsibility or trigger reassessment. Veterinarians may recommend reassessment or withdrawal, yet in numerous AAI programmes, the ultimate decision rests with programme coordinators or facilities. This pattern highlights a recurring ambiguity in the distribution of responsibility within the domain of AAI governance, a phenomenon that is echoed in welfare research conducted on the subject of Animal-Assisted Interventions [16,17,18]. The absence of explicit institutional frameworks results in the diffusion of accountability among handlers, facilities, and certifying veterinarians. Responsibility for welfare cannot rest with veterinarians alone; handlers, facilitators, hosting institutions, and AAI service providers play an equally fundamental role and must be integrated into the governance framework.

Poor welfare conditions may also pose risks to the human recipients of Animal-Assisted Interventions (AAIs). Animals exhibiting sub-optimal physical condition may demonstrate unpredictable behaviour, increasing the likelihood of defensive aggression or unreliability in performance of assigned tasks. In certain instances, these elements have the capacity to augment exposure pathways in environments where zoonotic agents are present, or in the event of the animals themselves being the source of the pathogens. These considerations provide an additional rationale for continuous welfare assessment and for allocating institutional responsibility for determining an animal’s ongoing suitability for participation.

From the perspective of relational theory, this institutional gap exemplifies what Fineman [6] identifies as the failure of law to account for the distribution of vulnerability. Unlike human workers, whose occupational exposure and burnout are regulated under labour law, AAI animals are not subject to analogous protections. The responsibility for this issue remains individualised and reactive, rather than collective and preventive. Such fragmentation has the potential to compromise animal welfare, human health, and professional integrity, thereby limiting veterinarians’ capacity to function as agents of systemic accountability rather than merely technicians. This observation is consistent with concerns raised in the AAI welfare literature, which emphasises the absence of mandatory review mechanisms and the reliance on variable, non-standardised oversight practices across programmes [15,16,17,19,20].

In the context of veterinary decision-making, relational accountability signifies that assessment and certification are not merely technical acts, but rather continuous interpretive processes informed by biological data, behavioural indicators, and institutional feedback. Ethical responsibility, therefore, arises through a dynamic interaction between evidence, context, and care. Within this paradigm, veterinarians are required to strike a balance between clinical expertise and relational ethics, undertaking a dual evaluation of animals’ changing vulnerability and responsiveness within the confines of institutional frameworks.

It can thus be concluded that a redefinition of veterinary responsibility grounded in relationships would reconceptualise the veterinarian not solely as a health certifier but as a contributor to the ethical integrity of institutions where Animal-Assisted Interventions are performed. Certification processes should be understood as ongoing, relational judgements embedded within adaptive medico-legal systems. Time-limited or conditional authorisations, for example, could require periodic re-evaluation based on behavioural and physiological indicators of stress, workload, and environmental suitability [4]. Furthermore, the integration of veterinary assessments with institutional welfare data could facilitate the identification of cumulative strain across facilities, thereby enhancing the effectiveness of monitoring and management strategies.

Moreover, the absence of proxy mechanisms for informed consent on behalf of animal participants remains an unresolved ethical problem. While animals cannot consent in the human sense, their behavioural and physiological responses can serve as indicators of willingness to continue or of a need for withdrawal. In contexts such as trauma therapy or palliative care, behavioural and physiological cues should be evaluated prior to the introduction of animals into situations that have the potential to elicit stress and subsequently monitored throughout the intervention. Because animals cannot give consent in the human sense, veterinarians could be empowered to interpret signs of willingness or reluctance as indicators of whether participation should continue or be halted [21].

Nevertheless, such behavioural indicators frequently necessitate longitudinal and context-specific observation that extends beyond what can be captured in a clinical consultation. Behaviour can vary considerably depending on the setting, the individuals involved, and the temporal rhythm of the intervention. Patterns of engagement, withdrawal, avoidance, or over-arousal frequently emerge only during the practical delivery of Animal-Assisted Interventions (AAIs) or during periods of rest. Consequently, the incorporation of systematic in situ and ex situ assessments by trained ethologists or behaviour specialists has the potential to serve as a valuable complement to veterinary evaluations, thereby providing a comprehensive documentation of behavioural patterns across various temporal and contextual domains. The integration of these complementary perspectives facilitates a more complete understanding of welfare and reinforces the institutional responsibility for continuous monitoring in AAI programmes.

Such mechanisms of preventive and ongoing assessments would serve to enhance the ethical coherence of AAI governance, thereby acknowledging animals as relational beings whose participation carries expressive significance.

### 3.2. Species, Selection and Age Regulation: Persistent Legal Blind Spots

Another critical gap lies in the insufficient regulation of species and age suitability for AAI participation. Many jurisdictions lack clear criteria for which species may ethically and legally engage in interventions, leading to inconsistent and sometimes exploitative practices. Although international bodies such as the International Society for Animal Assisted Therapy [22] and the International Association of Human–Animal Interaction Organizations [23] provide non-binding guidance restricting participation to domesticated species (e.g., dogs, cats, rabbits, horses, and donkeys), such recommendations have limited legislative force and are adopted unevenly across programmes.

Only a few national systems, such as Italy’s Linee guida nazionali per gli interventi assistiti con gli animali [24], formally codify positive lists of eligible species. However, even within these contexts, regulatory gaps persist, particularly for non-listed species employed in unregulated Animal-Assisted Activities rather than therapeutic programmes, where oversight is frequently absent. The resulting inconsistency may compromise both ethical and legal accountability, potentially leaving animals exposed to unsuitable conditions and institutions vulnerable to liability.

Furthermore, the age criteria for animals are seldom delineated with legal precision. Despite the existence of numerous professional guidelines advocating for the implementation of maturity thresholds (e.g., one to two years for dogs; one year for cats), these remain non-compulsory rather than mandatory [25,26,27]. However, developmental immaturity and age-related decline are critical dimensions of vulnerability, as both juveniles and geriatric animals exhibit species-specific risks that may reduce resilience during repeated interventions. From Fineman’s [6] perspective, vulnerability is dynamic and relational: both juvenile and geriatric animals experience heightened dependence and reduced resilience. Ignoring these life-course variables weakens the ethical coherence of AAI governance.

A further regulatory blind spot concerns the status of animals who are evaluated but ultimately not selected for AAI work. In numerous programmes, animals are sourced from existing companion-animal or equine contexts rather than being bred specifically for Animal-Assisted Interventions (AAIs). Consequently, non-selected animals are generally returned to their original role or are rehomed or sold in accordance with conventional practices for that species. However, there is a paucity of statutory or professional standards addressing the management of this transition, and the welfare implications vary considerably across species. While companion animals such as dogs can often be rehomed with relative ease, larger species, particularly horses, present more complex challenges due to their long-term care needs, management costs, and more limited rehoming options. These species-specific differences further highlight the inconsistency of current governance structures and the lack of guidance on institutional responsibility across the entire trajectory of AAI involvement.

### 3.3. Institutional Accountability and Systemic Oversight

Beyond individual veterinary or handler responsibility, the lack of institutional accountability, understood as the responsibility of the facility delivering the intervention or the regulatory authority overseeing AAI practice, constitutes perhaps the most significant blind spot in AAI regulation. Current frameworks rarely impose obligations on the institutions themselves (hospitals, schools, care facilities) that host AAI programmes. Welfare failures are regarded as isolated incidents rather than as systemic deficiencies in governance.

The relational theory posits a structural shift from individual to institutional responsibility. In the context of Animal-Assisted Interventions, this refers specifically to AAI service providers, such as hospitals, schools, therapeutic centres, and other organisations that deliver these programmes, who should be mandated to implement periodic welfare reviews, maintain records of animals’ workload and behavioural data, and establish mechanisms for reporting signs of distress or fatigue. The utilisation of inter-institutional monitoring platforms has the potential to enhance traceability across programmes, thereby mitigating the risk of overuse and cumulative stress [4]. Comparable monitoring frameworks have been developed in related domains, providing practical insights into the operationalisation of inter-institutional oversight. For instance, coordinated reporting systems are already in operation within public health surveillance networks, biosafety governance, veterinary public health for zoonotic-disease surveillance, and animal research oversight (e.g., IACUC-based reporting). The aforementioned systems are contingent on shared reporting standards and coordinated ethical review across multiple facilities. Collectively, these precedents illustrate that cross-facility monitoring is both viable and effective, thus providing a valuable analogue for the development of similar mechanisms in AAI settings.

This model mirrors the responsive state that Fineman [10] envisions, in which resilience is fostered through proactive, context-sensitive regulation. In the context of AAI settings, this necessitates the transformation of welfare oversight into a dynamic and distributed process, which is to be shared across veterinarians, handlers, administrators, and policymakers. Without such adaptive and institution-level accountability, even the most well-intentioned AAI programmes risk perpetuating the instrumentalisation of animals under the guise of therapeutic benefit.

### 3.4. Illustrative Case Studies and Policy Contexts

Across jurisdictions, general animal welfare legislation already recognises forms of shared or distributed responsibility for animal welfare. Within Europe, for instance, Germany’s *Tierschutzgesetz* and Austria’s *Bundesgesetz über den Schutz der Tiere (TSchG)* impose duties of care on anyone who keeps, handles, or is otherwise responsible for an animal. Switzerland adopts a similar approach through its Animal Welfare Act (*AniWA*) and the implementing Animal Protection Ordinance (AniPO), which extend legal obligations to all individuals involved in the keeping and supervision of animals, and require that their dignity and inherent worth be respected as far as possible. The United Kingdom’s *Animal Welfare Act 2006* likewise establishes a broad ‘duty of care’ applicable to any person responsible for an animal, regardless of ownership. Comparable principles also appear outside Europe; South Africa’s *Animals Protection Act 71 of 1962* places welfare obligations on any person who has possession or charge of an animal and Australian state and territory statutes similarly impose duties on persons in charge of animals to ensure appropriate care and to prevent unnecessary suffering.

These frameworks demonstrate that a baseline distribution of welfare duties across multiple actors is already present in many legal systems. However, they are not designed to regulate Animal-Assisted Interventions as structured institutional practices. They establish general obligations of care, but they do not typically address role-specific training, workload limits, programme-level welfare monitoring, or cross-institutional accountability; features that are central to the relational model developed in this paper.

Within this broader context, several existing guidance documents and organisational codes illustrate how relational governance can be implemented within Animal-Assisted Interventions (AAIs), even in the absence of dedicated legislation. However, it should be noted that, aside from Italy’s State–Regions Agreement adopting the National Guidelines for Animal-Assisted Interventions, there is still very little binding legislation specifically regulating Animal-Assisted interventions in most jurisdictions where such practices are formally implemented. As a result, internal policies, professional codes, and non-binding guidelines currently function as the de facto regulatory infrastructure in this area. The examples set out below are therefore not presented as substitutes for overarching legal frameworks, but as illustrations of how, in the absence of such frameworks, soft-law instruments have begun to fill this normative space and to articulate forms of distributed responsibility.

The Italian National Guidelines for Animal-Assisted Interventions [24] provide one of the most comprehensive legal frameworks in Europe. These guidelines explicitly recognise the shared responsibility of veterinarians, handlers, and institutional coordinators, and require that each AAI team include a veterinary professional responsible for continuous health and welfare monitoring. Although the guidelines remain principally procedural, they embody the principle of distributed accountability, which is a fundamental tenet of relational theory.

In Australia, the Animal Therapies Ltd. Code of Conduct [28] extends this relational approach by defining eligibility criteria for species, minimum age thresholds, and workload limitations for therapy animals. Importantly, the Code mandates ongoing assessment of animals’ behavioural suitability and emotional resilience, positioning the veterinarian as a central ethical agent within interdisciplinary teams. Such provisions reflect Fineman’s notion of institutional responsiveness, in which vulnerability is managed collectively rather than delegated to individuals.

At an international level, initiatives by the International Association of Human–Animal Interaction Organizations (IAHAIO) [23] and the International Society for Animal Assisted Therapy (ISAAT) [22] have helped to standardise welfare requirements across contexts. However, these instruments remain non-binding and rely on voluntary compliance. The embedding of such frameworks within national legislation would effect a transformation of soft ethical norms into tools of relational justice. Together, these examples show that the theoretical principles of relational autonomy and vulnerability can inform concrete regulatory practice, bridging the gap between bioethical ideals and institutional implementation.

These comparative frameworks collectively illustrate the feasibility of embedding relational principles into legal and ethical governance. The next section builds on these insights to propose an integrated model of relational accountability for Animal-Assisted. Interventions (AAIs).

## 4. Regulatory Horizons: Towards a Relationally Accountable Legal Framework

The preceding analysis reveals that the governance of Animal-Assisted Interventions (AAIs) is characterised by fragmented standards, discretionary practices, and limited institutional oversight. This phenomenon is indicative of a more profound conceptual deficiency, namely the inability to acknowledge animals as correlated subjects, beings whose ethical and legal status is derived from the relational dependencies, vulnerabilities, and institutional functions that define their participation.

Existing welfare models are predicated on the assumption of individual responsibility and episodic certification, thus offering reactive protection rather than continuous accountability. From a relational legal perspective, the requirement for justice is such that the realms of animal welfare, professional ethics, and institutional design must be integrated into a coherent regulatory architecture that recognises mutual interdependence.

### 4.1. Core Principles for Ethical–Legal Governance of Animal Assisted Interventions

A relationally accountable framework for Animal-Assisted Interventions (AAIs) can be structured around five interlocking principles derived from relational autonomy, vulnerability theory, and institutional ethics:

1. Relational Vulnerability

Animals’ welfare must be understood as co-constituted through their relationships with humans and institutions. Vulnerability is dynamic, shaped by species, age, workload, and environment—and thus requires ongoing, context-sensitive protection rather than static compliance [6,11].

2. Context-Sensitive Oversight

Regulation should adapt to species, setting, and function, recognising that an animal’s role in a psychiatric clinic differs from one in educational or rehabilitative contexts. A single welfare standard is insufficient; oversight must be proportionate to the intensity and relational complexity of the work.

3. Continuous Institutional Accountability

Institutions engaging in Animal-Assisted Interventions (AAIs) must bear continuing responsibility for animals’ welfare through regular reassessment, documentation of workload, and transparent reporting mechanisms. Oversight should shift from one-off certification to cyclical review, mirroring occupational-health systems for human professionals.

4. Transparency and Traceability

Shared data infrastructures should record the life-course of each participating animal—including health indicators, intervention frequency, and retirement. Traceability strengthens accountability and prevents the invisibility of welfare decline.

5. Reflexivity and Stakeholder Inclusion

The governance process should be characterized by participation, integrating veterinary, legal, ethical, and behavioural expertise alongside institutional stakeholders and animal-welfare representatives. Reflexive deliberation is a process by which ethical responsibility is distributed collectively and evolves over time.

It is evident that, collectively, these principles constitute a responsive model of governance, wherein law functions not solely as a constraint but rather as a system that actively fosters resilience and relational justice.

### 4.2. Institutional Innovations for Relational Governance

Translating these principles into practice requires institutional mechanisms capable of embedding ethical and legal accountability within everyday AAI operations.

The proposals introduced below operate at different temporal scales: some measures, such as the establishment of multidisciplinary oversight panels and retirement and transition protocols, can be implemented at facility or programme level in the short to medium term, whereas others, including ethical licensing systems in their formal regulatory sense, inter-institutional monitoring platforms, and specialised administrative agencies, constitute longer-term structural reforms requiring legislative or administrative redesign. Together, these proposals outline a multi-layered strategy capable of addressing both immediate operational needs and broader structural challenges.

- Specialised Administrative Agencies

Dedicated national or regional bodies should be established or existing animal-welfare authorities expanded with mandates to supervise AAI programmes. Their functions would include licensing, auditing, and enforcement of welfare standards, as well as coordination of veterinary, professional, and institutional data. The establishment of such agencies would serve to bridge the current gap between individual certification and public oversight [29].

- Ethical Licensing Systems

Authorisations for AAI work should become time-limited ethical licences, renewed only upon demonstration of continued compliance with welfare regulations and institutional transparency. This approach, informed by research-ethics frameworks, emphasises that ethical legitimacy is a continuous, evidence-based process rather than a one-time approval. It is important to note that the aforementioned licences would apply to AAI programmes and service providers, not to veterinarians, owners or animals themselves. Facilities seeking authorisation would be required to demonstrate adequate welfare safeguards, multidisciplinary oversight, and transparent reporting.

- Inter-Institutional Monitoring Platforms

A digital infrastructure for data sharing among veterinarians, institutions, and regulatory authorities could serve to record workload, intervention types, and welfare incidents, while also allowing for the real-time identification of cumulative stress or risk. Comparable systems of inter-institutional monitoring already exist in adjacent domains, such as animal research oversight, public health surveillance, and biosafety governance. These frameworks rely on shared reporting, harmonised standards, and coordinated ethical review across multiple facilities. It is posited that these models have the potential to inform the development of AAI-specific monitoring structures. The establishment of such an infrastructure would operationalise Fineman’s [19] vision of the responsive state, ensuring that vulnerability is monitored systemically rather than reactively.

- Multidisciplinary Oversight Panels

Each accredited provider should establish a standing ethics committee composed of veterinarians, animal-behaviour experts, legal scholars, and ethicists. The responsibility for reviewing cases that are borderline or complex, providing counsel on welfare interventions, and ensuring that the benefits to humans are proportionate to the burden on animals would fall upon the panels.

- Retirement and Transition Protocols

The formalisation of retirement procedures should encompass the establishment of criteria for the withdrawal from active work, with consideration given to factors such as age, health, and behavioural indicators. It is imperative that these transitions are institutionally supported, with an acknowledgement that ethical responsibility extends beyond the animal’s period of active service.

### 4.3. From Welfare to Relational Justice

These proposals represent a paradigm shift from a welfare-based model of protection to a relational conception of justice. Whereas welfare law traditionally safeguards animals from cruelty or harm, relational justice emphasises their embeddedness within interdependent systems that generate complementary responsibilities among the human actors involved in Animal-Assisted Interventions. In this model, veterinarians, institutions, and policymakers share an obligation to design environments that sustain animals’ well-being and support their relationally mediated responsiveness across time.

This reconceptualization also redefines the contours of professional ethics. Veterinarians become mediators of relational accountability, and institutions assume an active role in shaping conditions of care rather than functioning merely as service providers. Legal frameworks informed by relational theory thus help bridge the gap between ethical aspiration and enforceable obligation, aligning veterinary bioethics with contemporary theories of care and justice.

### 4.4. Future Directions and Policy Implications

Advancing relational governance of Animal-Assisted Interventions (AAIs) will require coordinated research and policy experimentation across several domains:

- Empirical and Longitudinal Welfare Studies: systematic data on workload, behavioural adaptation, and lifespan across species remain limited; longitudinal research is essential for evidence-based regulation.
- Comparative Legal Analysis: cross-jurisdictional studies can identify transferable models of adaptive licensing and welfare oversight.
- Institutional Design Research: the initiation of pilot programmes could facilitate the testing of the functionality of how licensing bodies and digital monitoring systems work within clinical and educational contexts.
- Normative and Theoretical Development: further integration of concepts such as vulnerability, care, and relational agency into bioethical theory will serve to refine the philosophical underpinnings of interspecies justice, thereby contributing to a more nuanced and comprehensive understanding of ethical principles in bioethical theory.

The transition towards relational accountability necessitates the acknowledgement that therapeutic benefits alone cannot justify the presence of structural opacity or excessive animal use. Ethical governance must reflect the mutual dependence of all participants, human and non-human, within therapeutic systems.

## 5. Comparative and Theoretical Outlook: Towards an Expanded Relational Jurisprudence

The proposal for a relationally accountable framework for Animal-Assisted Interventions (AAIs) gains depth when situated within broader international and interdisciplinary developments in animal law, bioethics, and environmental governance. Across jurisdictions and disciplines, a discernible shift is occurring from anthropocentric welfare regimes toward integrative approaches that recognise animals as sentient, relational, and institutionally embedded participants within multispecies communities.

### 5.1. Comparative Developments in Animal Law

Several jurisdictions have taken meaningful steps toward recognising animals as subjects of moral and legal consideration. France amended its Civil Code in 2015 [24] to define animals as *êtres vivants doués de sensibilité* (living beings endowed with sentience), thereby qualifying, but not abolishing, their property status (Code civil, Art. 515-14) [30]. Germany and Switzerland have long codified the moral significance of animals in constitutional or statutory provisions, mandating that their welfare be “protected as fellow creatures”. In 2021, Spain proceeded to reform its Civil Code [31], thereby acknowledging animals as sentient beings. The reform explicitly mandated that judicial decisions must take their welfare into account in cases involving ownership and custody.

Despite the largely symbolic nature of these reforms, they indicate a gradual shift in the civil law paradigm, moving away from a strict property-based framework. Civil-law systems often change slowly in this area because liability rules, property rights, and the legal status of animals are deeply embedded in longstanding doctrinal structures. Debates concerning ownership, compensation, and the scope of custodial duties generate strong divisions among scholars, which contributes to the persistence of traditional categories. In particular, the conventional linkage between dominion, control, and the allocation of risk has acquired a degree of conceptual inertia: adjusting one element would require reconfiguring the others, a move that many scholars perceive as destabilising for the overall architecture of private law. Moreover, debates concerning ownership, compensation, and the scope of custodial duties remain highly polarised, with no consensus on whether existing categories should be adapted, expanded, or replaced. This doctrinal contestation helps explain why even widely acknowledged shortcomings of the current framework do not readily translate into legislative reform. Against this background, most civil-law frameworks still stop short of articulating forms of institutional responsibility, the very dimension that relational theory highlights. The category of correlated subject proposed here fills this conceptual gap by providing an intermediate legal status: it recognises animals’ embedded agency and institutional vulnerability without relying on the full apparatus of legal personhood.

### 5.2. Relational Governance and the One Health/One Welfare Paradigm

In recent years, there has been growing recognition of the importance of the One Health and One Welfare paradigms, which integrate human, animal, and environmental well-being into a unified ethical and policy framework [32,33]. These approaches emphasise systemic interdependence and cross-sectoral accountability. However, the operationalisation of these concepts frequently remains technocratic, prioritising disease control or productivity over the moral architecture of interspecies relationships.

A relational jurisprudence, understood as a legal approach centred on relationships rather than individual subjects, enriches these paradigms by embedding the ethical dimension of care and responsiveness within their institutional design. In this paradigm, veterinarians and AAI institutions assume a pivotal role within an augmented One Welfare framework, accountable not solely for the mitigation of harm but also for the cultivation of conditions conducive to interspecies flourishing. This integration positions relational animal law within the broader context of global governance debates, aligning it with contemporary policy frameworks that have already been recognised by international bodies such as the World Organisation for Animal Health (WOAH) and the Food and Agriculture Organisation (FAO).

### 5.3. Multispecies Justice and Interspecies Solidarity

Parallel theoretical currents have emerged under the rubric of multispecies justice, a field that seeks to extend theories of environmental and social justice to include animals and ecosystems [34,35]. This scholarship insists that justice must account for the complex entanglements of species, institutions, and ecologies. Rather than treating animals as external to human society, it positions them as participants in shared systems of vulnerability and resilience.

The concept of correlated subjecthood resonates strongly with this perspective. It reframes animal agency not as autonomy in isolation but as relational participation shaped by institutional contexts and human norms. By aligning relational jurisprudence with multispecies justice, the paper contributes to a broader re-imagining of legal inclusion that situates AAIs as exemplary sites where interspecies solidarity can be institutionalised.

### 5.4. Toward an Expanded Relational Jurisprudence

Integrating insights from comparative law, One Welfare, and multispecies justice points toward a more ambitious ethical–legal horizon: an expanded relational jurisprudence capable of articulating responsibilities across species and institutional domains. Such a framework would move beyond binary categories of human and non-human, rights-holder and object, to recognise networks of care and dependency as constitutive of justice itself.

Within this paradigm, the veterinarian assumes the role of an institutional actor in the governance of interspecies relations, the AAI facility is established as a locale for ethical co-production, and the state emerges as a responsive guarantor of resilience across human and animal domains. This paradigm not only enhances the theoretical reach of animal law but also situates veterinary bioethics as a central discipline in designing equitable multispecies futures.

In this broader international context, the concept of correlated subjecthood also complements emerging global governance frameworks. Recent initiatives, including the European Union’s Animal Welfare Strategy 2030, the FAO’s One Welfare guidance, and the United Nations Environment Programme’s recognition of animal welfare as a dimension of sustainable development, underscore the necessity of integrated, cross-sectoral approaches. The integration of relational jurisprudence within these policy frameworks would serve to facilitate harmonised ethical and legal accountability for animals in AAIs, aligning them with the broader objectives of public health, sustainability, and social justice. This integrative perspective underscores the notion that interspecies justice is not merely a moral ideal, but rather a pragmatic necessity for coherent governance in an era of ecological interdependence. It also reinforces One Welfare’s emphasis on interdependent well-being across species and environments, providing conceptual continuity between clinical welfare practice, legal governance, and multispecies justice.

Recent scholarship has already further expanded this agenda. Coulter [36] emphasises the practical dimension of animal labour within interspecies policy, while Kymlicka and Donaldson [37] develop a comprehensive theory of interspecies justice that reinforces the need for institutional accountability across species lines.

#### Policy Implications

The following table (Table 1) summarises the main ethical and legal recommendations advanced in this paper and their potential policy applications within Animal-Assisted Interventions:

These recommendations operationalise the concept of correlated subjecthood by embedding relational ethics into practical governance structures. Together, they advance the shift from animal welfare to relational justice, ensuring sustained well-being for both human and non-human participants in Animal-Assisted Interventions (AAIs).

## 6. Conclusions

### 6.1. Conceptual Synthesis: From Welfare to Relational Justice

The analysis developed throughout this paper demonstrates that the governance of Animal-Assisted Interventions (AAIs) can no longer rely on a property-based or welfare-only conception of animal protection. Animals engaged in therapeutic, educational, and assistive work are not passive instruments, but rather context-dependent participants whose responsiveness is shaped and constrained by human-controlled environments.

The present study proposes a reconceptualisation of legal and ethical responsibility as distributed and relational rather than individual by bringing together Nedelsky’s [5] notion of relational autonomy and Fineman’s theory of universal vulnerability [6,10]. Autonomy is defined as a relational capacity sustained by care, responsiveness, and institutional design; vulnerability is defined as a shared condition that grounds obligations rather than diminishing moral worth.

Within this theoretical framework, animals involved in Animal Assisted Interventions (AAIs) are best understood as correlated subjects, defined by their embeddedness within institutional relationships of work, care, and dependency. This concept serves to bridge the prevailing gap between anthropocentric personhood and objectification, thus offering a middle path that acknowledges animals’ relationally mediated forms of responsiveness without necessitating full legal personhood. This paradigm shift in the conceptualization of animal protection transcends the confines of mere benevolence, thereby redefining it as a structural commitment to interspecies justice.

The integration of relational legal theory, veterinary ethics, and vulnerability jurisprudence thus provides a new conceptual architecture for animal law: one that recognises agency as co-produced, accountability as institutional, and justice as an ongoing practice of responsiveness.

This study contributes to ongoing debates in relational jurisprudence and bioethics, which increasingly call for deeper integration between animal law, veterinary practice, and institutional ethics. While scholars such as Deckha [7], Palmer and Sandøe [11], and Coulter and Blattner [12] have advanced the relational and vulnerability-based turn in animal ethics, this paper extends these insights to the concrete medico-legal and institutional dynamics of Animal-Assisted Interventions. In doing so, it bridges theoretical reflection and policy application, positioning veterinary professionals as pivotal agents of interspecies governance and situating animal-assisted practice within the broader agenda of multispecies justice and One Welfare.

### 6.2. Practical and Professional Implications

The practical implications of this reconceptualisation extend well beyond theoretical discourse.

For veterinarians, adopting a relational perspective entails a transition from technician to ethical co-governor of animal welfare within institutions. Certification and assessment become dynamic, context-sensitive acts of moral and legal responsibility. Veterinarians would not merely attest to an animal’s health status but participate in shaping institutional conditions—workload, rest periods, behavioural monitoring—that determine the sustainability of the animal’s participation. It is, therefore, recommended that continuous professional education in relational ethics and interspecies care should be integrated into veterinary curricula and continuing development frameworks.

For institutions, the relational model demands that animal welfare oversight be embedded into everyday governance institutions such as hospitals, schools, and rehabilitation centres that utilise Animal-Assisted Interventions (AAIs) are obliged to implement periodic welfare reviews, transparent record-keeping, and cross-institutional data-sharing. These measures would mirror human occupational-health frameworks, ensuring that risk, fatigue, and stress are recognised as institutional, not private, concerns.

For policymakers, relational accountability signifies a transition from regulatory minimalism to proactive stewardship. Rather than relying solely on welfare statutes or voluntary codes, states should support specialised agencies, ethical licensing systems, and inter-institutional monitoring platforms as outlined earlier. These tools translate moral theory into enforceable governance mechanisms capable of sustaining both animal and human well-being.

Finally, for the academic and professional community, the framework encourages a redefinition of disciplinary boundaries. The fields of veterinary science, animal law, and bioethics have historically been compartmentalised; however, a relational approach positions them within a unified epistemic ecosystem that emphasises interdependence, care, and institutional ethics.

### 6.3. Future Horizons: Research, Education, and Institutional Transformation

In order to achieve relational justice for animals in the context of Animal-Assisted Interventions (AAIs), there is a necessity for sustained empirical, normative, and educational engagement.

Empirical research should be conducted in order to map the lived experiences of working animals within a variety of institutional contexts, with the objective of identifying species-specific markers of stress, resilience, and adaptation. The utilisation of longitudinal data will facilitate the establishment of evidence-based welfare thresholds and time-bound participation models.

It is imperative that normative inquiry persists in its refinement of the philosophical foundations of interspecies justice. It is recommended that subsequent research endeavours explore the manner in which correlated subjecthood interacts with emerging fields such as multispecies justice, planetary health, and animal labour studies. The objective of this exploration is twofold: firstly, to gain a more profound comprehension of the manner in which legal systems might incorporate forms of relational agency and responsibility that extend beyond human boundaries; and secondly, to contribute to the theoretical framework of legal systems in a meaningful way by means of the insights derived from this exploration.

Education and training represent another area of exploration. The integration of relational ethics and institutional accountability into veterinary, legal, and policy education would equip professionals to engage ethically with the complexities of human–animal collaboration. The integration of interdisciplinary modules co-taught by veterinarians, legal scholars, and ethicists has the potential to cultivate a novel generation of professionals who exhibit proficiency in both empirical welfare assessment and normative reasoning.

At the institutional level, the path forward lies in the transformation of existing governance infrastructures into systems that are responsive and care-based. This necessitates not only the adoption of technical reforms but also the cultivation of organisational cultures that prioritise transparency, reflection, and ethical reciprocity. The notion of relational governance emphasises the necessity of humility and continuous learning, recognising that justice, akin to care, is inherently dynamic and perpetually negotiated within evolving multispecies relationships.

In conclusion, it is imperative that relational ethics be integrated within the governance framework of Animal-Assisted Interventions (AAIs), as it is imperative from both a moral standpoint and as a professional opportunity. The proposed model of veterinary and institutional practice is one that aligns compassion with accountability. Furthermore, it treats interspecies cooperation not as a site of exploitation, but as a shared project of resilience and well-being.

## Figures and Tables

**Table 1:** Key Components of a Relational Accountability Framework for AAIs (simplified version).

Domain	Core Elements
Veterinary Contribution	Welfare certification; assessment of health and basic behavioural indicators; contribution to continuous monitoring systems.
Behavioural and Ethological Input	In situ and ex situ behavioural assessment; observation of stress signals, engagement, avoidance, and recovery patterns across contexts.
Institutional Responsibilities	Record-keeping (workload, behavioural observations); periodic welfare reviews; internal reporting mechanisms; ensuring appropriate working conditions.
Oversight and Governance	Multidisciplinary oversight panels; clear allocation of responsibility; procedures for suspension or withdrawal from AAI work.
Regulatory Infrastructure	Ethical licensing of AAI programmes; auditing; harmonised welfare standards; national or regional coordination mechanisms.

Note: A detailed, expanded version of this table is available as Supplementary Table S1.

## Data Availability

No new data were created or analyzed in this study.

## Supplementary Materials

The following supporting information can be downloaded at: https://www.mdpi.com/article/10.3390/ani16010092/s1, Table S1: Summary of the relational governance principles proposed in this paper and their potential applications within Animal-Assisted Interventions. For readability, each principle is paired with one indicative mechanism and one expected outcome; expanded explanations are provided in the main text.
