# Supplementary material for: Correlated Subjects: Relational Ethics and Veterinary Legal Accountability in Animal-Assisted Interventions
Source: Animals (Basel). 2025 Dec 29;16(1):92. doi: 10.3390/ani16010092 (PMC12784780; doi:10.3390/ani16010092)
Supplement: Supplementary file 1 [file animals-16-00092-s001.zip › animals-4019909-supplementary.pdf]

Relational Principle	Proposed Mechanism/ Policy Tool	Expected Outcome
Relational Vulnerability	Continuous welfare assessment; dynamic certification protocols	Institutional recognition of evolving animal needs
Context-Sensitive Oversight	Species- and role-specific welfare standards	Regulation responsive to interspecies variation
Institutional Accountability	Mandatory record-keeping, workload monitoring, and welfare audits	Transparent and distributed responsibility
Transparency and Traceability	Shared databases across institutions and veterinary professionals	Prevention of overuse and cumulative fatigue
Reflexivity and Inclusion	Multidisciplinary ethics panels; stakeholder participation	Balanced decisions between human benefit and animal wellbeing
Retirement and Transition	Formalised withdrawal protocols and post-care support	Ethical closure and continued welfare post-service

Table S1. Summary of the relational governance principles proposed in this paper and their potential applications within Animal-Assisted Interventions. For readability, each principle is paired with one indicative mechanism and one expected outcome; expanded explanations are provided in the main text.
